# Mechanical Circulatory Support for End-Stage Heart Failure in Repaired and Palliated Congenital Heart Disease

**DOI:** 10.2174/157340311797484222

**Published:** 2011-05

**Authors:** Joseph B Clark, Linda B Pauliks, John L Myers, Akif Ündar

**Affiliations:** 1 Penn State Hershey Pediatric Cardiovascular Research Center, Departments of Pediatrics, Penn State Hershey Childrens Hospital, Penn State Hershey College of Medicine, 500 University Drive, Mail Code H085, Hershey, PA, 17033; 2 Surgery, Penn State Hershey Childrens Hospital, Penn State Hershey College of Medicine, 500 University Drive, Mail Code H085, Hershey, PA, 17033; 3 Bioengineering, Penn State Hershey Childrens Hospital, Penn State Hershey College of Medicine, 500 University Drive, Mail Code H085, Hershey, PA, 17033

**Keywords:** Mechanical circulatory support, Congenital heart disease, end-stage heart failure, pediatric heart transplantation, pediatrics, extracorporeal life support.

## Abstract

Approximately one in one hundred children is born with congenital heart disease. Most can be managed with corrective or palliative surgery but a small group will develop severe heart failure, leaving cardiac transplantation as the ultimate treatment option. Unfortunately, due to the inadequate number of available donor organs, only a small number of patients can benefit from this therapy, and mortality remains high for pediatric patients awaiting heart transplantation, especially compared to adults. The purpose of this review is to describe the potential role of mechanical circulatory support in this context and to review current experience. For patients with congenital heart disease, ventricular assist devices are most commonly used as a bridge to cardiac transplantation, an application which has been shown to have several important advantages over medical therapy alone or support with extracorporeal membrane oxygenation, including improved survival to transplant, less exposure to blood products with less immune sensitization, and improved organ function. While these devices may improve wait list mortality, the chronic shortage of donor organs for children is likely to remain a problem into the foreseeable future. Therefore, there is great interest in the development of mechanical ventricular assist devices as potential destination therapy for congenital heart disease patients with end-stage heart failure. This review first discusses the experience with the currently available ventricular assist devices in children with congenital heart disease, and then follows to discuss what devices are under development and may reach the bedside soon.

## INTRODUCTION

Although survival rates for patients with repaired or palliated congenital heart disease are now quite good, there remain a minority of patients who may experience either acute heart failure in the early postoperative period or end-stage heart failure much later [1]. Mechanical circulatory support can offer several important applications in the management of both acute and chronic heart failure following surgery for congenital heart disease [2-4]. Extracorporeal life support (ECLS) in the setting of congenital heart disease is most commonly applied in the early postoperative period for acute heart failure, and these patients are typically supported with extracorporeal life support systems, such as extracorporeal membrane oxygenation (ECMO). For patients with end-stage heart failure, the best definitive treatment is heart transplantation. For the repaired or palliated congenital heart disease patient with severe and intractable ventricular dysfunction, mechanical circulatory support can serve as a bridge to transplantation. However, the outcomes of ECLS for this indication are not as good as in respiratory failure or other emergency indications [5]. Relatively lower survival rates have been observed in patients who were younger and had cardiac failure, with recent survival rates of 39% for neonatal cardiac patients [6]. 

Against this background, ventricular assist devices (VADs) have experienced increasing use as a bridge to transplantation in children with encouraging results. Unfortunately, the number of potential heart transplant recipients far exceeds the donor pool, and heart transplantation cannot adequately meet the needs of this patient population [7]. Consequently, the development of cardiac assist devices for destination therapy represents an attractive future goal, and is the subject of ongoing research [8]. 

In adults, VADs have been used as bridge to recovery or transplant, and even as destination therapy. In adults with end-stage heart failure, VAD support has been associated with improved survival versus medical management [9]. Although clinical experience with VAD support of heart failure in congenital heart disease is still limited, an increase in VAD use in children has occurred since smaller devices became more widely available [10-13]. 

In 2010, Gazit and colleagues provided a comprehensive description and analysis of the options for mechanical circulatory support in children awaiting heart transplantation [14], and summarized their institution’s experience with the Berlin Heart as a bridge to transplantation [15]. In this review, we seek to provide an update on the most recent studies regarding pediatric mechanical circulatory support for children with congenital heart disease and end-stage heart failure, with a special focus on the options for long-term support, namely VADs and other systems. 

### Congenital Heart Disease: The Scope of the Problem 

Approximately one in one hundred neonates is born with congenital heart disease [16]. It is estimated that 0.4% of live-born infants require a heart operation or catheter-based intervention. The overall outlook has improved for these patients, with 85% of children born with congenital heart disease now surviving into adulthood [17]. During an earlier era when the mortality associated with reconstructive surgery was higher, the concept of primary heart transplantation for infants with complex disease was attractive, potentially offering improved survival compared to corrective surgery [18]. However, the availability of pediatric donor hearts never reached levels that could meet the needs of the population with congenital heart disease. For the last decade, the number of pediatric heart transplantations in the United States has remained essentially flat at less than 500 cases annually [19]. Meanwhile, surgical results for congenital heart disease have improved, and the vast majority of these patients can now be successfully managed with primary repair or staged palliation.

### End-stage Heart Failure in Congenital Heart Disease 

Despite advances in corrective and palliative surgery for congenital heart disease, some patients will develop acute or chronic end-stage heart failure and will require cardiac transplantation. These patients with congenital heart disease and end-stage heart failure fall into three broad categories. First, there are some infants without surgical options other than cardiac transplantation due to the complexity of their problem (e.g., unresectable cardiac tumor). Second, some patients reach the point of end-stage heart failure after one or more palliative operations which adversely affect outcomes [20]. It is estimated that approximately 10% of Fontan palliations experience early failure. There is some experience with heart transplantation after failed Fontan palliation of single-ventricle heart disease, and successful transplantation is possible after this type of palliation for single ventricle, albeit difficult [21]. Third, some patients with unrepaired congenital heart disease may survive well into adulthood but eventually progress to end-stage heart failure. For example, patients with congenitally corrected transposition of the great arteries (ventricular inversion) may present with failure of their systemic ventricle in fourth decade of life [22]. In this situation, the first operation for these patients may be cardiac transplantation. There is a recent report of successful palliation of three patients with congenitally corrected transposition of the great arteries with LVAD [23]. 

Another interesting aspect is that pre-treatment with ECLS may help to improve pulmonary hypertension in patients awaiting cardiac transplantation for congenital heart disease although it is unclear whether this improves the overall outcome [24-25].

Given the incidence of congenital heart disease, there will be a substantial number of patients with congenital heart disease in need of alternative treatment modalities. For instance, with approximately 4.3 million births in the United States in 2007 [26], it can be expected that more than 40,000 patients will be affected by congenital heart disease. It is thought that there are now close to 1,000,000 Americans with congenital heart disease. This value stands in stark contrast to the less than 500 pediatric cardiac transplantations performed annually [19]. Therefore, there is a vital need for alternative therapies for end-stage heart failure in this growing population.

## PEDIATRIC MECHANICAL CIRCULATORY SUPPORT: CURRENT DEVICES

### Berlin Heart EXCOR Pediatric

The Berlin Heart EXCOR Pediatric is the most common pediatric VAD for bridge to transplant in use today. Over 800 pediatric patients worldwide have been supported with a Berlin Heart as a bridge to cardiac recovery or transplant [http://www.berlinheart.de/index.php/newsroom/content/press_us/press_release_us]. While survival to transplantation is good (63%-89%) [15, 27-29], complications are also common in patients supported with the EXCOR device, such as thromboembolism (22%), bleeding requiring reoperation (29%), infection (67%), and adverse neurological events (69%) [15, 27-29]. Of these morbidities, the persistently high rate of stroke remains a troubling aspect associated with VAD support.

## PEDIATRIC VAD EXPERIENCE IN GERMANY AND EUROPE

Following the first implantation of the Berlin Heart EXCOR Pediatric at the German Heart Institute Berlin in 1990, this institution has accrued the largest single-center experience with the device worldwide. In 2006, Hetzer and colleagues reported their 15-year experience with the Berlin Heart EXCOR Pediatric [30]. In this report, the authors divided their experience into two time periods, and reported a number of salient findings. In the later era (i.e., 1999 to 2005), patients were more likely to be treated with a left VAD (LVAD) only, and had a higher survival to hospital discharge following either recovery or heart transplantation. Survival to discharge improved for both patients with cardiomyopathy (from 43% to 76%), and those with postcardiotomy heart failure (from 0% to 57%). Significant gains in outcomes were appreciated for the infants in the study, with higher rates of primary sternal closure, extubation, and survival. The authors attributed these improvements to earlier implantation of VADs, and a number of modifications both in the VAD circuit and in the management of anticoagulation. 

In 2010, Fan and colleagues from Berlin provided an update to their earlier experience with a focus on the support of young patients with the EXCOR Pediatric [31]. The authors reviewed a population of 56 small children with body surface area less than 1.2 m^2^ who were treated with VAD support from 1999 to 2009. Patients with cardiomyopathy accounted for the largest share (73%) of the group, with congenital heart disease comprising a lesser fraction (25%). This review noted a trend toward improved survival in patients treated only with an LVAD versus biventricular assist device (BiVAD) support. Consistent with an earlier report from Blume et al. [10]*,* significantly decreased survival was seen in patients with congenital heart disease.

In 2010, Brancaccio and colleagues also reported their experience with the Berlin Heart as a bridge to transplantation in small children [32]. They reviewed 10 children weighing less than 10 kg who were supported from 2002 to 2010, with a median age of 10.4 months and weight of 6.4 kg. ECLS was used in two patients prior to VAD support. An LVAD alone was used in seven patients, with a BiVAD in three. Half of the patients required at least one pump exchange. Three patients died of stroke, and one of sepsis. Five patients (50%) were successfully bridged to transplant, while the last was still awaiting transplant. Early post-transplant survival in this small cohort was 100% at a median of 7.5 months. These authors suggested that this type of mechanical support can be an effective bridge to transplantation strategy, but is currently associated with significant morbidity and mortality. 

## PEDIATRIC VAD EXPERIENCE IN NORTH AMERICA

Although the Berlin Heart EXCOR came into routine clinical use in 1992 at the German Heart Institute Berlin [30], the device was not available in the United States until a decade later. Although the first implantation of the EXCOR in the United States occurred in 2000, widespread use was not seen until 2004 [11]. Prior to this time, VAD use in children in North America was primarily limited to older, larger patients who could be supported with the smallest adult-size VADs, such as the Thoratec VAD. Consequently, support options for infants and small children were usually limited to ECLS or centrifugal assist systems. 

In 2006, Blume and colleagues [10] reported on the growing use of VADs in children as a bridge to heart transplantation. During a ten-year time frame ending in 2003, ventricular assist devices were implanted in 99 (4%) of 2375 children listed for heart transplantation. Over the course of the study, there was a three-fold increase in the use of VADs, with devices present at transplant in 2.3% of patients in the early cohort but in 7.2% of patients in the late cohort. In this study, patients supported with VADs were more likely older (median 13.3 versus 4.8 years), larger (median 56 versus 20 kg), and less likely to have congenital heart disease (22% versus 60%) than non-VAD patients. Two short-term devices were used: the Bio-Pump (Medtronic, Minneapolis, MN) in 16, and the BVS 5000 (Abiomed Inc., Danvers, MA) in 10. Four long-term devices were used: the Thoratec VAD in 53 patients, a Heartmate LVAS (Thoratec Corp., Pleasanton, CA) in 13, a Novacor LVAS (WorldHeart Inc., Oakland, CA) in 3, and the Berlin Heart EXCOR Pediatric in 1. The major diagnostic group was comprised of patients with cardiomyopathy (78%), with congenital heart disease accounting for the remainder (22%). Risk factors for death while awaiting a transplant included earlier era of implantation, female gender, and diagnosis of congenital heart disease. 

In 2008, Davies and others provided a follow-up to the work of Blume et al. by examining the effect of type of mechanical circulatory support on post-transplantation survival [33]. Using data from the United Network for Organ Sharing, these authors studied 2532 transplantations in children less than 19 years of age from 1995 to 2005. Mechanical circulatory support was present at the time of transplantation in 431 patients (17%), and included VADs in 241 (9.5%), ECLS in 171 (6.8%), and intra-aortic balloon pumps in 19 (0.8%). Dilated cardiomyopathy accounted for the majority of patients receiving mechanical support, with congenital heart disease accounting for a minority (29%). When mechanical support was implemented, patients with cardiomyopathy were more often supported with VADs (n=180) than ECLS (n=66). On the contrary, patients with congenital heart disease were more commonly supported with ECLS (n=90) and less so with VADs (n=33). Compared to the VAD patients, ECLS patients were both younger (3.8 versus 12.2 years) and smaller (16.8 versus 52.9 kg). These authors showed that 5- and 10-year post-transplantation survival was better for patients supported with VADs than for those supported with ECLS, and that late survival was similar between patients receiving VADs and those who did not require mechanical support. Although ECLS was associated with decreased long-term survival, this effect was primarily exhibited in the early postoperative period. ECLS patients had higher rates of end-organ failure, and consequently had a higher early mortality rate. Beyond this initial postoperative period, the survival curves for all the patient groups were parallel. The association of ECLS with higher early mortality was independent of the diagnosis (e.g., cardiomyopathy versus congenital heart disease). This study made no distinction regarding the type of VAD, and did not report on any specific devices. The authors proposed that some of the smallest patients who were classified in the VAD cohort were likely supported with centrifugal assist devices and not with pneumatic pulsatile assist devices. These small patients with a body surface area less than 0.3 m^2^ were found to have a higher long-term mortality. The authors suggested that these patients, if they had in fact been supported with centrifugal devices, may not have received some of the benefits seen with the pulsatile VADs, such as higher rates of extubation and mobilization, and that these factors may have contributed to the poorer outcomes in these patients.

In 2009, Imamura and colleagues from Arkansas reported their experience with mechanical circulatory support as a bridge to transplantation [34]. They reviewed patients from 2001 to 2008 who received mechanical support as a bridge to transplant. The patient cohorts were evenly distributed between ECMO (n=21) and Berlin Heart EXCOR (n=21). The overall incidence of congenital heart disease was lower (24%) than the incidence of either cardiomyopathy (50%) or myocarditis (26%). Although there was a trend toward younger age in the ECMO group compared to the VAD group (2.1 versus 4.1 years, p=0.07), the groups were found to be otherwise similar in weight, etiology of heart failure, and degree of co-morbidity. Despite mortality in the first 5 consecutive patients supported with VADs, a survival advantage was demonstrated for VAD support compared to ECMO. Survival to transplant, recovery, or continued support was 57% in the ECMO group and 86% in the EXCOR group. These authors suggested that the presence of the learning curve in this series may actually lead to an underestimation of the survival benefit of VAD support. EXCOR use was also associated with significantly longer periods of support versus ECMO (mean 42 versus 15 days). Unfortunately, both modalities were associated with significant neurologic morbidity, with equivalent stroke rates of 38%. One important decision associated with the implementation of VAD support is the choice of device implantation in one or both ventricles. These authors treated all patients who were transitioned from ECLS with biventricular devices. All remaining patients (who had not been on ECLS) received an LVAD alone, and no patient later required the addition of a right VAD. These authors suggested that ECMO and VADs should not be viewed as competing technologies, but rather as complementary therapies. Pointing to utility of ECMO, the authors cited the typically wider availability and greater experience with ECLS, especially for emergency support and for use in small infants. For example, in this study, one third of patients included in the VAD cohort had been initially supported with ECMO, thus making ECMO a bridge to VAD. In 2010, Stein and colleagues from Stanford updated their experience with pediatric VAD support as a bridge to transplantation, offering the largest United States single-center report to date [12]. Using data drawn from the Interagency Registry for Mechanically Assisted Circulatory Support, they reported on outcomes for 25 children less than 18 years old who were supported with VADs from 1998 to 2007. Patients with congenital heart disease comprised a small fraction of the population (16%), with various forms of cardiomyopathy accounting for the remainder. ECMO support preceded VAD implantation in 5 (19%) of patients. The most commonly used devices were the Thoratec pediatric VAD in 16 (59%) and the Berlin Heart EXCOR in 9 (33%), consistent with a slightly older and larger patient population with median age of 12 years and weight of 47 kg. While overall survival to transplantation was encouraging at 74%, considerable morbidity was appreciated, with over half of the patients having the events of respiratory failure, major infection, major bleeding, hepatic dysfunction, and right heart failure. Additionally, neurologic morbidity was highlighted by a major stroke rate of 48% and a dysfunction rate of 59%. Neurologic events were the most common cause of mortality, accounting for 3 of the 7 deaths in the study. Going forward, the larger unanswered question, according to the authors, remains when to initiate VAD support in children. As suggested by others [30], earlier initiation of VAD therapy may improve survival, as patients receive support prior to the onset of vital organ dysfunction or failure. On the other hand, premature use of VAD therapy may unnecessarily expose some children to the risks and morbidities that accompany this type of support, as some patients may have survived to transplantation without device implantation. According to the authors, this question of timing of VAD support presents an ongoing important and challenging clinical scenario.

In 2010, Humpl and colleagues from Toronto reported their experience with Berlin Heart [27]. From 2004 to 2008, 15 patients with an average age of 8.8 years and weight of 31.1 kg were supported with the device. Dilated cardiomyopathy was the predominant diagnosis (n=14, 93%), with congenital heart disease in one patient with a single ventricle Glenn circulation. ECMO was used initially in 3 patients (20%) prior to VAD support. All patients were supported with biventricular devices, with the exception of the single ventricle patient who received a single VAD. Adverse neurologic events were seen in 3 patients (20%). No patients were weaned to recovery, and two patients (13%) died on while on VAD support. Survival to transplant was seen in 13 patients (87%), including the patient with congenital heart disease. 

In 2011, Morales et al. reported on the initial North American experience with the Berlin Heart EXCOR Pediatric [11]. Prior to the start of the Berlin Heart investigational device exemption trial in 2007, nearly 100 devices were implanted at centers in the United States and Canada. These authors attempted to aggregate this early experience, and provide an overview of its efficacy as a bridge to transplant. Data was available for 73 of 97 patients covering a period from 2000 to 2007. As in other reports, congenital heart disease was seen in a minority of patients (26%), while dilated cardiomyopathy was the most common cause of heart failure (58%). As a cohort, these patients were small, reflecting the niche of the Berlin Heart, with a median patient age of 2.1 years and weight of 11 kg. ECMO provided a bridge to VAD support in 22 patients (30%). A majority of patients were treated with the LVAD alone (n=42, 57%), while BiVADs were used in the remainder. Younger age and biventricular support were risk factors for mortality while awaiting transplantation. Survival to transplantation was seen in 51 patients (70%) and survival to recovery in 5 (7%). Contrary to other reports [10, 31], congenital heart disease was not found to be a risk factor for death on mechanical support. Additionally, the use of ECMO prior to VAD implantation was also not associated with increased mortality. Although this report represents the largest single-device experience for pediatric VAD support in North America, the authors acknowledge that limited conclusions can be drawn from this incomplete clinical data. For example, no morbidity data was available, precluding any comment about the rates of brain injury, thromboembolic complication, infection, or device failure. With this type of morbidity data being gathered in the current Berlin Heart clinical trial, results regarding safety and efficacy will be forthcoming. In the meantime, these authors concluded that the Berlin Heart VAD is likely a feasible therapy as a bridge to transplantation. 

## PEDIATRIC VAD SUPPORT OF THE SINGLE VENTRICLE PATIENT

Some authors have reported that they do not offer VADs to patients with single ventricle congenital heart disease [34], instead opting to support those children with ECLS. Nevertheless, there are a handful of case reports from the last decade that describe the use of VAD support for single ventricle heart failure. VanderPluym and others provided such a case report as well as a review of the literature regarding the use of ventricular assist devices in pediatric patients with univentricular hearts [35]. These authors described VAD support of a 3-year-old with early heart failure following a Fontan operation. The patient’s anatomy was revised back to a bidirectional cavopulmonary Glenn connection, with initiation of ECMO. Support was later converted to a Berlin Heart EXCOR Pediatric device which was implanted with systemic right ventricular and aortic cannulation. Following nearly six months of VAD support, this patient underwent successful hear transplantation. As these authors acknowledge, the paucity of published reports on VAD support in single ventricle patients precludes firm recommendations regarding surgical management strategy. With this caveat, the authors proposed the following techniques for VAD support of palliated univentricular hearts. For patients with shunt dependent circulation (e.g., a Norwood patient with a Blalock-Taussig shunt), adequate support should be feasible with systemic ventricular inflow and aortic outflow. However, due to the parallel circulations and pulmonary runoff, higher pump flow rates will likely be required. Similarly, patients with a bidirectional cavopulmonary connection (e.g., a Glenn or Hemi-Fontan patient), may be adequately supported with the same cannulation strategy employing systemic ventricular inflow and aortic outflow. Finally, VAD support of the failing Fontan can be managed in several ways, depending on the anatomy and physiology of the specific patient. In the case of pump failure due to ventricular dysfunction, VAD support with systemic ventricular inflow and aortic outflow may be sufficient. However, if elevated pulmonary vascular resistance leads to systemic venous hypertension and right-sided failure, then a second VAD may be required to support flow through the pulmonary circulation. In this situation, separation of the two circulations is performed by takedown of the Fontan pathway. 

## PEDIATRIC MECHANICAL CIRCULATORY SUPPORT: DEVICES UNDER TESTING OR DEVELOPMENT

### Maquet Cardiohelp

The Maquet Cardiohelp is a compact, portable extracorporeal life support system. It has been used with the Maquet Quadrox-i adult oxygenator to provide emergency life support due to cardiogenic shock during patient transport. All components of this circuit are integrated, but changing the oxygenator to a neonatal or pediatric version (*e.g.,* Maquet Quadrox-i neonatal or Quadrox-i pediatric) could expand the usage of this device to pediatric patients. The adult circuit has a maximum flow rate of 7 L/min, which limits the upper bound on flow rates in potential pediatric patients to the maximum of the chosen oxygenator. However, this system is currently designed only for short-term use, and would require investigations into mechanical issues associated with longer use in order to make the transition to pediatric applications [http://www.maquet-cardiohelp.com/cardiohelpsystem/introduction.html].

### CircuLite Synergy System

Originally intended for partial support in adult patients, the CircuLite Synergy System (CircuLite, Inc., Saddle Brook, NJ) is an implantable micro-pump with a maximum capability of 3 L/min. Due to its small size and compatible flow rates, CircuLite was awarded a National Institutes of Health Fast-Track Phase I-II Small Business Innovation Research grant in July 2009 to convert their adult micro-pump into a pediatric specific pump capable of complete unloading of the heart. In preliminary juvenile bovine studies, the CircuLite Synergy System provided adequate left ventricular unloading, normal end-organ function, and no device malfunctions at a two-week time point post-implantation [36].

### Medos Deltastream DP3

An improvement to the previous Medos Delastream DP2 (MEDOS Medizintechnik AG, Stolberg, Germany), this new diagonal flow rotary pump is approved in Europe for up to 7 days of continuous use. In addition to the ability to generate both pulsatile and non-pulsatile flow, the DP3 pump also has a low priming volume (16 mL) and very fine control over low flow rate adjustments, making it suitable for neonatal use [37]. Preclinical studies in adult sheep demonstrated the ability of the DP3 pump to run for 7 days without incident in 5 out of 6 animals [38]. Initial clinical results show low thrombogenicity and hemolysis associated with the pump [37].

### Ventracor Ventrassist

Currently undergoing clinical trials, the Ventrassist pump (Ventracor, Inc., Sydney, Australia) is a third generation, implantable LVAD with a range of 1.5-10 L/min. Currently, it is being investigated for bridge to transplant in adult patients. In 98 adult patients, 85% and 82% of patients were alive on support or transplanted at 6 and 12 months post-implantation [http://www.ventracor.com]. Due to its larger size, this device is not currently feasible for use in neonates, but larger pediatric patients may benefit from the future application of this device.

### Abiomed Impella 5.0

The Abiomed Impella 5.0 (ABIOMED, Inc., Danvers, MA) is a minimally invasive catheter pump that can generate flows of 5 L/min for up to 7 days. It is currently approved by the United States Food and Drug Administration (FDA) as an investigational device and is available in Europe under CE Mark approval. In Europe, it has been used in more than 250 adult patients, providing support for post-cardiotomy cardiogenic shock or as a bridge to transplant. The low technical requirements of this device make it ideal for use in high-risk patients and those with multi-organ dysfunction, which would make para-corporeal implantable LVADs unsuitable. Several reports of adult patients in refractory cardiogenic shock have shown that use of the Impella 5.0 helped restore ventricular and end-organ function, and contributed to successful bridging of those patients to longer-term mechanical circulatory support devices [39].

The previous model Impella 2.5 was evaluated in small lambs to assess the feasibility of the catheter pump design for use in pediatric patients. Five out of the six lambs survived to two-weeks and the authors concluded that the implanted system generated stable flow at pediatric flow rates for 14 days, while maintaining hematologic stability with minimal tissue injury due to the implantation procedure [40]. In 2009, one pediatric patient with refractory cardiogenic shock due to viral myocarditis was stabilized with this device and successfully bridged to recovery [41].

## THE PUMPS FOR KIDS, INFANTS, AND NEONATES (PUMPKIN) PROJECT

The National Heart, Lung, and Blood Institute (NHLBI) recently awarded four grants for the development of new pediatric life support pumps, under the PumpKIN program. The goal of this program is to develop small, implantable pumps for use in small pediatric and neonatal patients. The current grant awardees include: LaunchPoint Technologies, Inc, Pittsburgh, PA and consortium; Ension, Inc., Pittsburgh, PA; University of Maryland School of Medicine; and Jarvik Heart, Inc., New York, NY.

LaunchPoint Technologies and its collaborators at the University of Pittsburgh are designing the PediaFlow Pediatric VAD. Their newest version, the PF3, is attempting to meet the design requirements of full implantability in children 3-15 kg, flow rates of 0.3-1.5 L/min, and be sustainability of 6 months of circulatory support, while minimizing hemolytic trauma, thrombosis, and infection [www.launchpnt.com].

Jarvik Heart, in collaboration with the University of Maryland and others, is redesigning the Jarvik 2000 axial flow pump for pediatric use. In juvenile sheep experiments, it was shown that the pediatric Jarvik 2000 could pump 1.4-2.5 L/min of blood for up to 70 days without important hemolysis or end-organ damage [42]. The infant Jarvik 2000 model is still under development.

Both the Ension pCAS system and Pedi PumpLung from the University of Maryland will be self-contained devices capable of pumping and oxygenating blood simultaneously [43]. It remains to be seen whether either device will be provide comparable capability to the Maquet CardioHelp currently in use.

### Penn State Hershey Pediatric ECLS System

Perfusion quality is of the utmost importance to neonatal and pediatric patients undergoing ECLS. The minimization of post-ECLS complications, including neurological and other vital organ injury, depend on adequate perfusion of vital tissues. To this end, the faculty, perfusionists, and nurses at the Penn State Hershey Pediatric Cardiovascular Research Center have designed a new neonatal and pediatric ECLS circuit, whose components have been selected based on rigorous studies conducted by our center [44-50]. This new circuit has many advantages over previous systems, including: reduced priming time and volume, smaller size, significantly reduced pressure drop (~90% lower) through the oxygenator, and the ability to be staffed by a nurse instead of a dedicated respiratory therapist. We have already trained 110 clinicians from five institutions in 2010, as well as our own in-house staff. 

## CONCLUSIONS

Patients with congenital heart disease and end-stage heart failure currently have a limited number of options for long-term mechanical circulatory support. In recent years, significant advances have been made, and more devices are under development with some having already reached pre-clinical trials. Over the next few years, continued improvements in the field of pediatric mechanical circulatory support are expected, including the addition of new pediatric VADs, new magnetically levitated centrifugal pumps, and new low pressure drop hollow-fiber membrane oxygenators for ECLS. 

## Figures and Tables

**Fig. (1):**
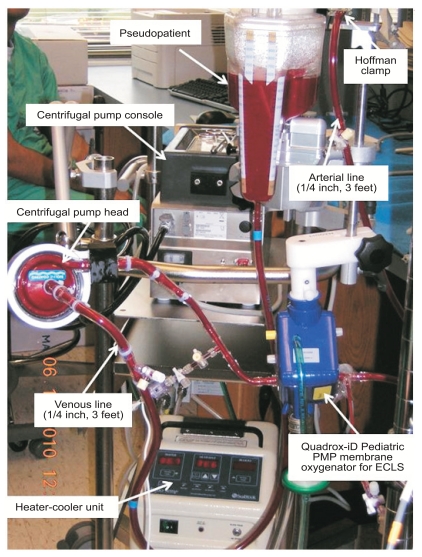
The Penn State Hershey ECLS circuit.

**Fig. (2):**
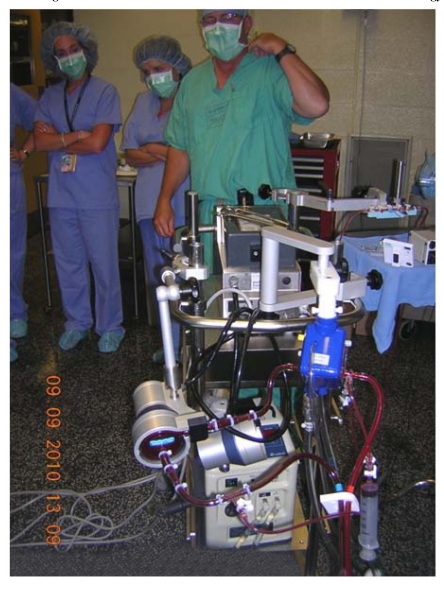
Training session using a piglet model.
